# Prevalence of Human Papillomavirus in Self-Taken Samples from Screening Nonattenders

**DOI:** 10.1128/JCM.00550-17

**Published:** 2017-09-25

**Authors:** J. U. H. Lam, M. Rebolj, D. M. Ejegod, H. Pedersen, C. Rygaard, E. Lynge, E. Harder, L. T. Thomsen, S. K. Kjaer, J. Bonde

**Affiliations:** a Department of Pathology, Copenhagen University Hospital Hvidovre, Hvidovre, Denmark; b Clinical Research Centre, Copenhagen University Hospital Hvidovre, Hvidovre, Denmark; c Department of Public Health, University of Copenhagen, Copenhagen, Denmark; d Unit of Virus, Lifestyle and Genes, Danish Cancer Society Research Center, Copenhagen, Denmark; e Department of Obstetrics and Gynecology, Copenhagen University Hospital Rigshospitalet, Copenhagen, Denmark; Johns Hopkins University School of Medicine

**Keywords:** HPV assays, HPV self-sampling, nonattenders, primary screening

## Abstract

The Copenhagen Self-Sampling Initiative (CSi) has shown how human papillomavirus (HPV)-based self-sampling can be used to increase screening participation among 23,632 nonattenders in the Capital Region of Denmark. In this study, we describe HPV prevalence and genotype frequency in 4,824 self-samples as determined by three HPV assays (the CLART, Onclarity, and Hybrid Capture 2 [HC2] assays) and compare the results with those for physician-taken follow-up samples. The HPV self-sample findings were also compared to the findings for a reference population of 3,347 routinely screened women from the Horizon study, which had been undertaken in the same screening laboratory. Nonattenders had an HPV prevalence of 11.3% as determined by the CLART assay, which was lower than that for women from the Horizon study (18.5%). One-third of the CSi women who tested HPV positive by self-sampling tested HPV negative on the physician-taken follow-up sample. The CLART and Onclarity assays agreed on 64% (95% confidence interval [CI], 60 to 68%) of the HPV-positive self-taken samples. When the HC2 assay results were added into a three-way comparison, the level of agreement decreased to 27% (95% CI, 24 to 29%). Our findings suggest that further validation of HPV assays on self-taken samples is needed for optimal HPV detection and correct clinical management of HPV-positive women.

## INTRODUCTION

Human papillomavirus (HPV)-based self-taken samples can be an alternative to cervical cytology screening for women who do not attend screening (nonattenders) (1, 2). A meta-analysis showed that with specific combinations of collection devices and HPV assays, the accuracy of detection of high-grade cervical intraepithelial neoplasia (≥CIN2) was similar to that with physician-based sampling (3). Most self-sampling studies to date have used the Hybrid Capture 2 (HC2) assay for HPV testing (2, 4–11), but currently, no single HPV assay is recognized as the gold standard. On routine screening with physician-taken samples, assays are considered validated if they show noninferiority to the HC2 assay or GP5+/6+ PCR, the two assays that were the basis for four European randomized trials (12). However, no guideline exists on the criteria for the use of HPV assays with self-taken samples.

The pilot implementation of “The Copenhagen Self-Sampling Initiative” (CSi) was initiated to investigate whether self-sampling by nonattenders was a viable option for increasing participation in screening. Another aim was to compare three HPV assays on self-taken samples. By use of an opt-in strategy, 20% of 23,632 invited nonattenders returned self-taken samples (13). All samples were tested in a split-sample protocol with the CLART HPV2 (Genomica, Madrid, Spain) and BD Onclarity HPV (BD, Sparks, MD, USA) tests. Moreover, a limited subset of 1,008 samples was also tested using the HC2 assay (Qiagen, Hilden, Germany), which was the routine assay in the laboratory at the time the pilot started. Women with a positive result on any of the HPV assays were recommended to have a physician take a cytology sample, to be cotested for cytology and HPV, as follow-up.

The frequencies of different HPV genotypes among self-sampling nonattenders are infrequently reported in the literature, mainly because the majority of the studies used the HC2 assay, which does not allow one to distinguish between the HPV genotypes detected. In this study, we not only employed assays with extended or full genotyping but also compared the findings to those of the Danish Horizon study, a previous study using physician-taken samples from routinely screened women, where the CLART assay was also used in that study.

One of the major outcomes of the Horizon study was that the frequencies of ≥CIN2 detection were fairly similar for the assays compared, but the assays detected different HPV infections in the same samples (14). On samples from approximately 3,000 women undergoing primary screening, only 27% of all infections detected by four commercially available assays were confirmed by all assays, and when the assays were compared pairwise, only half of all HPV infections were confirmed on both assays. No similar HPV assay performance issues on self-taken samples have been described earlier.

In this study, we compared the detection of HPV infections by three assays (CLART, Onclarity, and HC2) and the subsequent cytology results for women with detected infections. The findings were compared to those for routine screening samples from the Horizon study (15, 16).

## RESULTS

High-risk HPV (HR-HPV) infections were detected in 769 (15.9%) self-taken samples, based on a positive test result on any assay (where testing by the HC2 assay was undertaken only on the first 1,008 samples). The Onclarity assay showed the highest prevalence (14.1%), followed by the CLART and HC2 assays (11.3% and 7.2%, respectively) (Table 1). The HPV prevalence detected in self-taken samples appeared to be lower than that for physician-taken samples in the Horizon study (18.5%) when tested by the CLART assay. This was also the case with the age-specific prevalences. The prevalence of HPV appeared not to be related to the women's screening history. Of the 769 women who tested positive for HPV, 641 went for follow-up (follow-up compliance rates were 83% for all ages, 90% at the ages of 27 to 29 years, 82% at 30 to 39 years, 84% at 40 to 49 years, 77% at 50 to 59 years, and 87% at 60 to 65 years [data not tabulated]). Almost one-third of the women with physician-taken follow-up samples after a positive self-taken sample had a diagnosis of atypical squamous cells of undetermined significance or higher-grade lesions (≥ASCUS), a proportion 2-fold higher than that for women in the Horizon study with a positive HPV test at baseline.

**TABLE 1:** Prevalence of high-risk HPV in 4,824 self-taken samples from the CSi pilot, as detected by the Onclarity, CLART, and HC2 assays, by age, screening history, and subsequent cytology test results for HPV-positive women, compared with results for 3,347 women undergoing primary cytological screening in the Horizon study

Characteristic	No. (%) of self-taken primary screening samples in the CSi^*a*^					No. (%) of physician-taken primary screening samples in the Horizon study	
	Onclarity and CLART			HC2			
	Total tested	HPV positive by Onclarity	HPV positive by CLART	Total tested	HPV positive	Total tested	HPV positive by CLART
Age group (yr)							
27–29	383 (100.0)	98 (25.6)	82 (21.4)	63 (100.0)	15 (23.8)	478 (100.0)	165 (34.5)
30–39	1,080 (100.0)	205 (19.0)	170 (15.7)	201 (100.0)	19 (9.5)	1,295 (100.0)	273 (21.1)
40–49	1,200 (100.0)	142 (11.8)	124 (10.3)	255 (100.0)	22 (8.6)	897 (100.0)	116 (12.9)
50–59	1,265 (100.0)	138 (10.9)	105 (8.3)	279 (100.0)	11 (3.9)	462 (100.0)	45 (9.7)
60–65	896 (100.0)	97 (10.8)	63 (7.0)	210 (100.0)	6 (2.9)	215 (100.0)	19 (8.8)
Total	4,824 (100.0)	680 (14.1)	544 (11.3)	1,008 (100.0)	73 (7.2)	3,347 (100.0)	618 (18.5)
Screening history^*b*^ for women aged ≥34 yr							
Long-term unscreened	1,599 (100.0)	190 (11.9)	147 (9.2)	337 (100.0)	19 (5.6)	133 (100.0)	20 (15.0)
Intermittently screened	2,416 (100.0)	287 (11.9)	229 (9.5)	525 (100.0)	28 (5.3)	2,158 (100.0)	287 (13.3)
Total	4,015 (100.0)	477 (11.9)	376 (9.4)	815 (100.0)	47 (5.8)	2,291 (100.0)	307 (13.4)
Physician-taken cytology samples from HPV-positive women							
Normal	457 (71.3)	384 (68.8)	318 (66.9)		50 (73.5)		509 (82.4)
≥ASCUS	182 (28.4)	172 (30.8)	156 (32.8)		18 (26.5)		109 (17.6)
Inadequate	2 (0.3)	2 (0.4)	1 (0.2)		0 (0.0)		0 (0.0)
Total	641 (100.0)	558 (100.0)	475 (100.0)		68 (100.0)		618 (100.0)

a CSi, Copenhagen Self-Sampling Initiative; HC2, Hybrid Capture 2 assay. Invalid test rates were 3.9% (*n* = 191) and 0.5% (*n* = 23) for the Onclarity and CLART assays, respectively. By definition, the HC2 assay reports no invalid test results. Three samples (0.1%) were inadequate for HPV testing by both the CLART and Onclarity assays. Because HPV results were required to be reported to women within 10 days of the arrival of the sample in the laboratory, women received their results based on the CLART and HC2 assays only for the 191 samples that were invalid by Onclarity.

b Long-term unscreened, no cytology sample registered in the Patobank in ≥10 years; intermittently screened, one or more cytology samples registered within the past 10 years.

HPV16 was the genotype most frequently observed both for routinely screened women and in self-sampling, constituting 25.4% and 24.3%, respectively, of the HR-HPV types (data not tabulated), and with a detected prevalence of 2.9% in self-sampling. The second most frequent HR-HPV genotype in self-sampling was HPV51 (2.0%), followed by HPV31 (1.6%) (Table 2). In the Horizon study, the most frequent genotypes were HPV16 (4.5%), HPV31 (2.8%), and HPV52 (3.2%). Infections with more than one HPV genotype were observed in approximately 56% of all HPV-positive samples in the Horizon study, whereas for self-sampling, the prevalence of multiple infections was below 48% (data not tabulated). The numbers and proportions of women with high- and low-risk infections are shown in Table 3.

**TABLE 2:** Distribution of high-risk HPV genotypes among women undergoing self-sampling in the CSi study, compared with that for routinely screened women participating in the Horizon study, by use of the CLART assay

High-risk HPV genotype	Total no. (prevalence [%]) of infections^*a*^ in the following study:	
	CSi^*b*^ (*n* = 4,801)	Horizon^*c*^ (*n* = 3,336)
HPV16	138 (2.9)	150 (4.5)
HPV18	29 (0.6)	57 (1.7)
HPV31	76 (1.6)	95 (2.8)
HPV33	30 (0.6)	67 (2.0)
HPV35	46 (1.0)	42 (1.3)
HPV39	18 (0.4)	20 (0.6)
HPV45	23 (0.5)	39 (1.2)
HPV51	97 (2.0)	79 (2.4)
HPV52	74 (1.5)	107 (3.2)
HPV56	19 (0.4)	35 (1.0)
HPV58	52 (1.1)	91 (2.7)
HPV59	42 (0.9)	49 (1.5)
HPV68	5 (0.1)	55 (1.6)

a The numbers of infections with the different genotypes do not add up to the total numbers of women with high-risk HPV infections (544 in the CSi study and 618 in the Horizon study) because some women had multiple HPV infections.

b Samples from 4,824 women were tested by the CLART assay. Of these, 4,801 (99.5%) had valid results.

c Samples from 3,347 women were tested by the CLART assay, and 3,336 (99.7%) had valid results.

**TABLE 3:** Numbers and proportions of women with high- and low-risk HPV infections in the CSi and Horizon studies

Patient group	No. (%) of women with infections detected in the following study:	
	CSi	Horizon
Total with high-risk HPV infections	544 (11.3)	618 (18.5)
A single high-risk HPV infection	284 (5.9)	272 (8.2)
Multiple infections, including ≥1 high-risk HPV genotype	260 (5.4)	346 (10.4)
Total with low-risk HPV infections^*a*^	618 (12.9)	375 (11.2)

a The CLART assay detected 1 or more of the 22 low-risk genotypes but no high-risk HPV infection.

**FIG 1:**
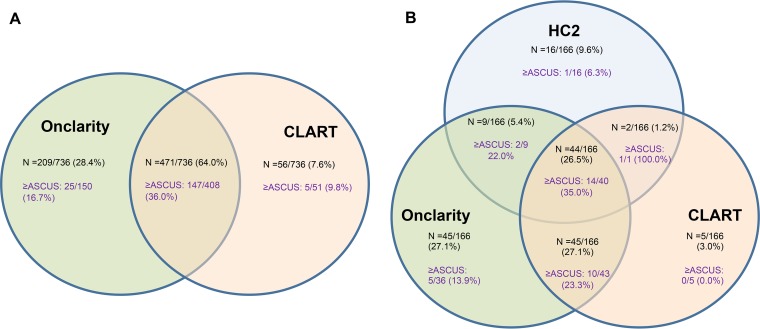
Agreement of the HC2, Onclarity, and CLART assays on the detection of HPV in self-taken samples and cytology diagnoses from follow-up samples. Note that ≥ASCUS was determined on physician-taken follow-up samples by cytology, and the different denominators reflect the completeness of follow-up in each subgroup. (A) In total, 4,612 samples gave valid results on the Onclarity and CLART assays. Of these samples, 736 (16.0%) were positive by at least one of the two assays, and 609 (81%) of the women with positive results had a follow-up cytology registered. (B) In total, 964 samples gave valid results on all three assays (HC2, Onclarity, and CLART). Of these, 166 (17.2%) were positive by at least one of the assays, and 150 of the women with positive results (90.4%) had a follow-up cytology registered.

Of 736 HR-HPV infections detected by the Onclarity or CLART assay in the CSi study, a total of 471 infections (64% [95% confidence interval {CI}, 60 to 68%]) were detected by both assays (Table 4; Fig. 1A). Women for whom infections were detected by both assays were more likely to have a diagnosis of ≥ASCUS.

**TABLE 4:** Agreement between the CLART and Onclarity assays on self-taken samples^*a*^

Type of infection	Total no. (%) positive by:		Total no. of infections	No. of samples:			% positive agreement (95% CI)
	Onclarity	CLART		Positive by both assays	Positive by CLART, negative by Onclarity	Negative by CLART, positive by Onclarity	
By definition of a positive CLART test result^*b*^							
≥1 of 13 high-risk genotypes	680 (14.7)	527 (11.4)	736	471	56	209	64.0 (60.4–67.5)
≥1 of 13 high-risk genotypes or HPV66	680 (14.7)	573 (12.4)	748	505	68	175	67.5 (64.0–70.9)
By genotype^*c*^							
HPV16	149 (3.2)	135 (2.9)	162	122	13	27	75.3 (67.9–81.7)
HPV18	46 (1.0)	28 (0.6)	47	27	1	19	57.4 (42.1–71.7)
HPV31	89 (1.9)	76 (1.6)	94	71	5	18	75.5 (65.6–83.8)
HPV45	58 (1.3)	23 (0.5)	58	23	0	35	39.7 (27.0–53.3)
HPV51	73 (1.6)	93 (2.0)	100	66	27	7	66.0 (55.8–75.2)
HPV52	72 (1.6)	70 (1.5)	81	61	9	11	75.3 (64.5–84.2)

a Of 4,824 samples, 4,612 (95%) had valid HPV results on both assays.

b For management purposes, and in line with the latest IARC definitions, we assumed that the CLART assay detected high-risk HPV infections if it detected one or more of the 13 high-risk genotypes (HPV16, -18, -31, -33, -35, -39, -45, -51, -52, -56, -58, -59, and -68). According to the manufacturer's settings, the Onclarity assay returns a positive result if 1 or more of the 13 high-risk genotypes or HPV66 is detected (14 genotypes in total).

c Comparison of genotype agreement was possible only with 6 HPV types, because the Onclarity assay reports the remaining 8 targeted genotypes (HPV33, -58, -35, -39, -68, -59, -39, and -66) in combination.

The relatively low agreement was not substantially increased when genotype HPV66 was included on the CLART assay so that it would match the genotypes targeted by the Onclarity assay. The levels of agreement were highest for HPV16, HPV31, and HPV52 (all ∼75%), whereas the lowest level of agreement was observed for HPV45 (40%) (Table 4).

When the HC2 assay was added into the comparison, with 964 valid test results, the level of positive agreement was 27% (Fig. 1B). In pairwise comparisons, the level of agreement between the CLART and HC2 assays was 38%, and that between the Onclarity and HC2 assays was 33%. The detected prevalence of HPV infections in this subset was similar to that in the entire study (14.8% by the Onclarity assay, 10.0% by the CLART assay, 7.4% by the HC2 assay, and 17.2% by any of the three assays).

Almost two-thirds of women with HPV-positive test results were also found HPV positive in the follow-up sample (69.1% for the CLART assay and 63.7% for the Onclarity assay) (Table 5). Women who came early for follow-up had a slightly lower HPV prevalence on the follow-up sample than women who came later, although the difference was not statistically significant (*P* = 0.67) (data not reported).

**TABLE 5:** High-risk HPV prevalence agreement between self-taken samples and physician-taken follow-up samples stratified by follow-up time^*a*^

Test and follow-up time for self-taken samples	No. of physician-taken follow-up samples received	No. of physician-taken samples positive for high-risk HPV by the same assay	High-risk HPV prevalence agreement (%) between self-taken samples and physician-taken follow-up samples (95% CI)
CLART^*b*^			
<27 days	279	188	67.4 (61.5–72.9)
≥27 days	174	125	71.8 (64.5–78.4)
Total, all samples	453	313	69.1 (64.6–73.3)
Onclarity^*c*^			
<27 days	326	196	60.1 (55.8–64.3)
≥27 days	206	133	64.6 (54.6–71.1)
Total, all samples	532	339	63.7 (59.7–68.0)

a The follow-up time was the number of days between the date on which the HPV result on the self-taken sample was sent to the woman and the date on which the follow-up cytology sample was registered in the Patobank. The median follow-up time was 27 days.

b The CLART assay detected high-risk HPV infections in 544 of 4,824 self-taken samples (11.3%). Of these, 453 (83.3%) had a valid HPV test result on the physician-taken follow-up sample.

c The Onclarity assay detected high-risk HPV infections in 680 of 4,824 self-taken samples (14.1%). Of these, 532 (78.2%) had a valid HPV test result on the physician-taken follow-up sample.

The use of the cycle threshold (*C_T_*) values of the internal controls included in the Onclarity assay, as well as the *C_T_* values for positive samples, allowed a quantitative evaluation of sample quality. Mean *C_T_* values on self-taken samples were approximately 2 cycles lower than those on physician-taken samples for all three human beta-globin (HBB) control wells (mean differences, −2.00 with HBB1, −1.99 with HBB2, and −1.99 with HBB3), indicating a larger concentration by volume of human material in self-taken samples than in physician-taken samples (Table 6). Discrepant sample sets with HPV-positive self-taken samples but HPV-negative physician-taken samples showed higher genotype-specific *C_T_* values on the self-taken sample than did the concordant sample sets (by 2 to 5 cycles) (Table 7). Concordant sample sets tended to have similar genotype-specific signal strengths. Finally, *C_T_* values tended to be lower in self-taken samples found positive by all three HPV assays than in self-taken samples that were positive only by the Onclarity assay (difference in median signal strengths, about 8 cycles) (Table 8).

**TABLE 6:** Comparison of *C_T_* values for human beta-globin by the Onclarity assay on self-taken and physician-taken follow-up samples^*a*^

HBB type^*b*^ (no. of valid results)	*C_T_* (95% CI)		Mean difference^*c*^ (95% CI)
	Self-taken samples	Physician-taken follow-up samples	
HBB1 (496)	21.85 (21.68–22.02)	23.85 (23.71–23.98)	−2.00 (−2.19, −1.83)
HBB2 (500)	21.83 (21. 67–22.00)	23.83 (23.69–23.97)	−1.99 (−2.17, −1.81)
HBB3 (498)	21.65 (21.49–21.81)	23.66 (23.53–23.79)	−1.99 (−2.17, −1.82)

a Of the 4,824 women with a self-taken sample, 680 tested HPV positive by the Onclarity assay. Of these, 504 (74.1%) women had a follow-up sample with a valid result. *C_T_*, cycle threshold.

b HBB, human beta-globin. Onclarity has three controls, in three different bulks, for human beta-globin material (HBB1, HBB2, and HBB3). For eight women, the *C_T_* value was reported as zero for either the self-taken sample or the follow-up sample with HBB1, but valid *C_T_* values were returned with HBB2 and HBB3, leaving 496 pairs of valid *C_T_* values for the analysis. Four samples with HBB2 and six samples with HBB3 failed to yield valid results.

c Calculated by subtracting the *C_T_* for the physician-taken sample from the *C_T_* for the self-taken sample.

**TABLE 7:** Comparison of genotype-specific signal strengths,^*a*^ measured by the Onclarity assay, on self-taken samples and physician-taken follow-up samples^*b*^

HPV genotype	Median (IQR) *C_T_* for samples with the following results^*c*^:			
	Brush positive, physician negative (*n* = 165)		Brush positive, physician positive (*n* = 339)	
	Self-taken samples	Physician-taken samples	Self-taken samples	Physician-taken samples
HPV16	32.47 (30.27–33.29)	Negative	26.47 (23.49–29.99)	25.34 (22.49–30.19)
HPV18	30.57 (28.45–31.88)	Negative	27.46 (23.85–29.81)	27.90 (23.95–29.81)
HPV31	30.66 (27.67–31.94)	Negative	25.85 (22.65–30.30)	26.65 (23.18–29.65)
HPV33/58	28.11 (26.93–31.23)	Negative	28.26 (24.74–30.61)	26.76 (24.04–29.92)
HPV35/39/68	30.65 (25.40–31.72)	Negative	26.44 (24.87–30.46)	27.14 (24.71–30.70)
HPV45	29.55 (27.59–32.90)	Negative	27.33 (23.66–31.56)	29.88 (25.69–31.88)
HPV51	30.45 (26.76–32.84)	Negative	25.75 (22.79–30.18)	28.45 (24.96–32.84)
HPV52	30.00 (28.96–31.34)	Negative	26.04 (22.02–28.73)	26.31 (18.97–30.81)
HPV56/59/66	30.75 (28.81–32.66)	Negative	26.04 (21.73–29.52)	27.21 (23.59–30.44)

a Expressed as the cycle threshold (*C_T_*). The cutoff was a *C_T_* value of 34.2.

b In total, of the 532 women with HPV infections detected by the Onclarity assay on the self-taken sample who had follow-up physician testing, 504 (95%) had a valid Onclarity test result on the physician sample.

c IQR, interquartile range. “Brush positive,” HPV-positive test results for self-taken samples; “physician negative,” HPV-negative test results for physician-taken follow-up samples.

**TABLE 8:** Median signal strength^*a*^ on samples with HPV infections detected by one assay, two assays, or three assays^*b*^

Assay(s) by which samples were found HPV positive	No. of samples	Median (IQR) *C_T_* value
One assay (Onclarity)	45	31.42 (29.80–33.28)
Two assays		
Onclarity and CLART	45	30.05 (28.58–31.72)
Onclarity and HC2	9	25.45 (20.22–25.89)
All three assays (Onclarity, CLART, and HC2)	44	23.47 (21.50–24.91)

a *C_T_* value on the genotype-specific well with the strongest signal.

b For 964 samples, all three assays (Onclarity, CLART, and HC2) gave valid results. In total, 166 samples (17.2%) were HPV positive on at least one of the assays (Fig. 1). Of these, 143 (86.1%) were positive by Onclarity, with *C_T_* values available for reporting.

## DISCUSSION

### General findings.

Among self-sampling attenders, the prevalence of HR-HPV was between 11% (by the CLART assay) and 14% (by the Onclarity assay), and about one-third of HPV-positive women had abnormal cytology upon follow-up. Agreement in the detection of HR-HPV between the CLART and Onclarity assays was observed in approximately two-thirds of cases overall, and in three-fourths of cases when HPV16, HPV31, or HPV52 infections were involved. About one-third of the women whose self-taken samples tested HPV positive were found to be HPV negative on the physician-taken follow-up sample, and this was particularly the case when the signal strength in the self-taken sample was close to the manufacturer-set cutoff, indicating small amounts of viral input material. The detected HR-HPV prevalences, overall and genotype specific, were lower than those in a routinely screened population from the same uptake area, although among the infected women, the proportion with abnormal cytology was higher.

### Strength and limitations.

Both the CSi and the Horizon study were population-based studies undertaken in a routine screening laboratory. Our laboratory is the sole provider of cervical screening for this part of Denmark; it has a long-standing cervical screening program with a ∼75% coverage rate (17). Hence, our findings are representative for the general population in a real-world setting, even though the laboratory's catchment area during the Horizon study included only the urban Copenhagen and Frederiksberg municipalities, whereas during the CSi study, the catchment area had been expanded to include the periurban areas of the entire Capital Region (including the former Copenhagen, Frederiksborg, and Bornholm counties besides the two municipalities). Moreover, the same senior laboratory staff participated in the two studies, ensuring the same standards for both studies. Finally, since our laboratory is the principal site for the validation of the Onclarity assay on SurePath samples (18), we have several years of experience in operating the Onclarity assay and instrumentation.

A limitation of the comparisons between the HC2, Onclarity, and CLART assays in the CSi may be that BD's Cervical Brush Diluent (CBD) medium, which was used to resuspend the self-sampling brush heads prior to analysis, has not been used for self-sampling previously, although it has been used extensively on physician-taken screening samples (18). The CLART assay is a well-described assay with good performance compared to the HC2 assay on both SurePath (19) and ThinPrep (20) samples and has been used for self-sampling in our laboratory previously (21).

### Clinical implications and comparison with other studies.

This is one of the first studies comparing different HPV assays in terms of their ability to detect HR-HPV in self-taken samples. Besides, this is an unsupervised self-sampling setting, which is the most relevant setting for a routine rollout of this service to screening nonattenders.

In line with previously published experience on physician-taken samples (14, 22), we found the positive agreement between the Onclarity and CLART assays to be just above 60%. This is comparable to the detection of HPV in routinely screened women, where pairwise agreement in a screening population reached 58% for the CLART and cobas assays (14). The agreement with the HC2 assay in this self-sampling population was below 40%, and the detected HPV prevalence was also lower on the HC2 assay than on the other two assays. Whereas the majority of European self-sampling studies in well-screened populations used only the HC2 assay for the detection of HPV infections (2, 4–8, 10), Enerly and colleagues (21) used the HC2 and CLART assays to investigate HPV prevalence among 169 women who returned a self-taken sample for HPV detection. In this small study, with the HPV analyses performed in our laboratory, the positive agreement for the assays was still only 50%. The disagreement is mainly technology driven (14), but our results underline the need for validation criteria for HPV testing on self-taken samples to facilitate a minimum of quality assurance.

For screening purposes, the relevant endpoint is histological confirmation of precancerous high-grade cervical lesions especially; this is beyond the scope of the current analysis and will be reported in detail separately.

One-third of the women found HPV positive on the self-taken sample were found HPV negative on the follow-up physician-taken sample. This discrepancy is highly interesting and relevant to the future implementation of self-sampling. One explanation for this observation could be spontaneous HPV clearance, yet the same decrease in HPV detection in the physician-taken sample was also seen in women who came for follow-up within 1 month after returning the self-taken sample. Alternatively, some women may have inadvertently sampled the vaginal canal, whereas the physicians are taught to visualize the cervix prior to taking the screening sample. Finally, self-taken samples were preprocessed in 3 ml CBD, whereas the physician-taken samples were suspended in 10 ml SurePath. The higher concentration of biological material per volume unit in a processed self-taken sample may lead to higher signal strengths (i.e., lower *C_T_* values) for real-time PCR and a higher proportion of samples above the cutoff for HPV positivity. The *C_T_* values on positive self-taken samples of women whose physician-taken samples were also HPV positive tended to be lower than those for women whose physician-taken samples were HPV negative. Finally, stronger signals were found when all three assays confirmed the HPV-positive test result than when only one assay detected the infection. In line with our research on routinely screened women from the Horizon study, this could also suggest that women whose HPV infections had higher signal strengths may have been more likely to harbor high-grade CIN (16). Nevertheless, this finding remains to be confirmed for women undergoing self-sampling.

### Conclusion.

Cervical cancer screening of nonattenders who participated in self-sampling yielded an overall HPV prevalence of 16%, detected by any of the three HPV assays used, which was somewhat lower than the prevalence detected among women attending routine screening in the same screening laboratory. Self-sampling women, however, had a higher proportion of cytological abnormalities upon follow-up. As in routine screening, self-taken samples showed substantial discordance between assays detecting HPV infections.

## MATERIALS AND METHODS

### Study design.

**FIG 2:**
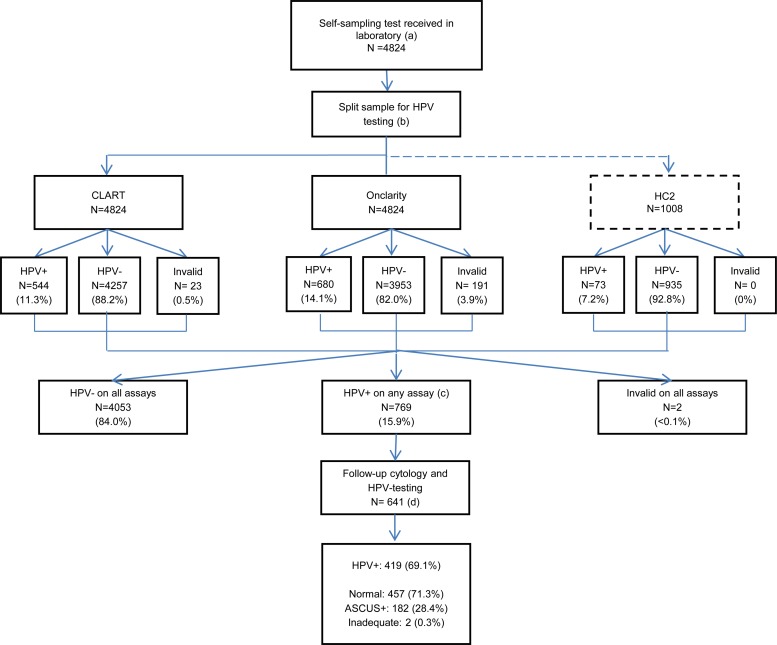
Flow chart of the study design: HPV prevalence and follow-up cytology. (a) A total of 23,632 nonattenders from the Capital Region were invited to be screened via self-sampling; of these, 4,824 (20%) accepted the offer and returned a self-sampling test. (b) Self-taken samples were aliquoted into four split samples. The first three aliquots were for HPV testing, and the fourth (not shown) served as backup material and constituted a biobank. (c) All women with detected HPV infections were recommended to see their GP for a cytology sample. Compliance to follow-up was 83.3% (641/769). (d) By mistake, 35 of the 641 (5.3%) follow-up samples were not tested for HPV, but they all underwent cytology evaluation.

In Denmark, women are invited for screening every 3 years (at the ages of 23 to 49 years) or every 5 years (at the ages of 50 to 65 years). The design of the CSi study has been explained elsewhere (13). Briefly, 23,632 nonattenders (defined as having had no screening for ≥4 years [at ages 27 to 49] or ≥6 years [at ages 50 to 65]) from the Capital Region were invited to order a self-sampling brush (Evalyn; Rovers, Oss, the Netherlands). In total, 4,824 (20%) women returned the self-taken sample. Each brush was preprocessed and was aliquoted into four split samples (Fig. 2). For clinical management within the study, a woman was considered HPV positive and recommended to undergo physician-administered sampling if any of the three assays detected high-risk HPV (HR-HPV). Women were notified of their results by letter, and the result was also sent to the general practitioner (GP) if consent was given by the woman. The subsequent cytology follow-up samples were cotested for HPV and used for triage to colposcopy. Women who were normal by cytology, regardless of the HPV test result, were recommended for cytology and HPV cotesting after 12 months. Women who had >ASCUS, regardless of the HPV test result, were referred directly to colposcopy, which was also the case for women who had ASCUS and were HPV positive. Women who were HPV negative but had ASCUS were recommended to have a cytology retest after 6 months. The cytotechnician was not blinded to the woman's HPV status. Women with no HPV infections detected in their self-taken sample were recommended to participate in the next round of routine screening.

The Horizon study was undertaken in the same screening laboratory in 2011 (14–16, 23–28), and from this study, we included 3,347 women (27 to 65 years old) undergoing primary screening. Their physician-taken SurePath samples were tested by the HC2, CLART, Aptima (Hologic, San Diego, CA, USA), and cobas (Roche, Rotkreuz, Switzerland) assays. Concurrent cytological diagnoses were known for all samples, and the cytotechnician was blinded to the HPV test results.

### Preprocessing of self-sampling brushes.

All returned brushes were preprocessed and were analyzed for HR-HPV. Brush heads were removed from the holders and were placed in empty 5-ml Eppendorf tubes (see Text S1 in the supplemental material). Three milliliters of BD's Cervical Brush Diluent (CBD) was added. Brush heads were removed after 15 min, and samples were vortexed to achieve homogeneous mixtures. Each sample was aliquoted into four empty tubes using the following schedule: 450 μl was transferred to an Eppendorf tube for DNA extraction and CLART analysis, 1,000 μl to a tube for Onclarity testing, and 500 μl to a tube for HC2 analysis (the first 1,008 brushes), while all residual material was stored at −20°C for backup.

### HPV DNA testing.

For the CLART assay, two 200-μl aliquots were transferred directly to a MagNA Pure 96 system (Roche Diagnostics, Mannheim, Germany) for DNA purification. The two 100-μl purified DNA outputs were then pooled. The assay provided full genotyping of 35 HPV types, without quantitative measurement of signal strengths. For clinical management, HR-HPV types were defined as HPV16, -18, -31, -33, -35, -39, -45, -51, -52, -56, -58, -59, and -68, corresponding to the latest classification of genotypes as carcinogenic (group 1) or probably carcinogenic (group 2A) by the International Agency for Research on Cancer (IARC) (29). Low-risk types were defined as HPV6, -11, -26, -40, -42, -43, -44, -53, -54, -61, -62, -66, -70, -71, -72, -73, -81, -82, -83, -84, -85, and -89, corresponding to the IARC definition as possibly carcinogenic (group 2B) or low-risk (not classifiable as carcinogenic [group 3]) genotypes.

For the Onclarity assay, the 1,000-μl aliquot was prewarmed for cell lysis. The sample was loaded into the Viper Lt real-time PCR system (BD, Sparks, MD, USA). The Onclarity assay detects six HR-HPV genotypes individually (HPV16, -18, -31, -45, -51, and -52), whereas another seven HR-HPV genotypes and one low-risk HPV genotype are detected in three combinations (HPV33/58, HPV35/39/68, and HPV56/59/66). The standard cycle threshold (*C_T_*) cutoff for HPV positivity is 34.2.

For the HC2 assay, DNA denaturation was performed manually on the 500-μl CBD medium aliquot before testing. HPV testing was performed on the automated Rapid Capture system (Qiagen, Gaithersburg, MD, USA), and the results were read automatically on a luminometer. The threshold for positive test results was 1 relative light unit (RLU)/cutoff (CO). Due to budgetary restrictions, HPV testing by the HC2 assay was completed only on the first 1,008 consecutive self-samples.

### Data sources.

Women's screening histories since January 2000 were retrieved from the nationwide Pathology Database (Patobank), which has been virtually complete for the entire country since the mid-2000s.

The invitations for self-sampling were sent between May 2014 and April 2015. For the current analysis, we retrieved the screening activities until November 2015, which allowed for delays in returning the brush (13), the laboratory testing and reporting, and the cytology follow-up.

Women were categorized as “intermittently screened” if they had a cytology sample within the last 10 years before the self-sampling invitation and as “long-term unscreened” if they had no sample for ≥10 years. The analyses using this categorization were restricted to women ≥34 years old, i.e., those who had been eligible for screening for ≥10 years.

### Statistical analyses.

Due to hardware failure and technical issues with the Viper Lt system, some of the samples were stored at −20°C before testing with the Onclarity assay. No significant differences were seen when HPV prevalences determined for frozen and fresh samples were compared, including a comparison of the respective *C_T_* values and adjustment for age (data not shown). Hence, we included all test results in the analyses.

As in the Horizon study (14), positive agreement between assays was calculated as the proportion of samples testing positive on any assay that were confirmed by all assays compared.

For the comparison of positive HPV test results in self-taken samples and physician-taken follow-up samples, we calculated medians and interquartile ranges (IQR) for the signal strengths (*C_T_* values) of the Onclarity assay. The *C_T_* medians were stratified according to the median follow-up time (27 days) for women who went for follow-up.

The 95% confidence intervals (CIs) for proportions and for prevalence agreement were calculated on the assumption that they were binomially distributed, whereas the 95% CIs for mean differences in *C_T_* values were calculated by taking into account that the observed differences were normally distributed.

Stata SE 13.1 and Microsoft Excel 2010 were used for data analyses.

### Ethical approval.

The CSi was a time-limited pilot implementation initiative mandated by the Danish Health Authority. The Horizon study was undertaken as a quality development study in accordance with the Committee Act under The National Committee on Health Research Ethics (2011 §14, part 3). In both cases, therefore, formal ethical approval was not required. Linkage to the Patobank was approved by the Danish Data Protection Agency under notification numbers AHH-2015-084-04139 and AHH-2015-080-04109 for the CSi and Horizon studies, respectively.

## Supplementary Material

Supplemental material
