# Supplementary material for: Prevalence of Human Papillomavirus in Self-Taken Samples from Screening Nonattenders
Source: J Clin Microbiol. 2017 Sep 25;55(10):2913–23. doi: 10.1128/JCM.00550-17 (PMC5625377; doi:10.1128/JCM.00550-17)
Supplement: Supplemental material [file supp_55_10_2913__index.html]

Supplemental material 

# Prevalence of Human Papillomavirus in Self-Taken Samples from Screening Nonattenders

## Supplemental material

- Supplemental file 1 -

  Text S1 (Protocol for pretreatment of Evalyn dry brush for HPV DNA testing)

  PDF, 66K
