# Supplementary material for: Prevalence of Human Papillomavirus in Self-Taken Samples from Screening Nonattenders
Source: J Clin Microbiol. 2017 Sep 25;55(10):2913–23. doi: 10.1128/JCM.00550-17 (PMC5625377; doi:10.1128/JCM.00550-17)
Supplement: Supplemental material [file JCM.00550-17_zjm999095645s1.pdf]

10 April 2017

Supplementary file 1: PROTOCOL - PRETREATMENT OF EVALYN DRY BRUSH FOR HPV-DNA TESTING

Utensils/instruments:

- 5 mL empty Eppendorf tubes
- 1.5 mL empty Eppendorf tubes
- Empty BD VIPER tubes
- Sterilized tweezers
- Vortexer, 3000 rpm

Reagents:

- 3 mL BD CBD media

Procedure:

- Remove brush head from holder with tweezer and place it in an empty 5 mL Eppendorf tube. Add 3 mL BD CBD media.
- Vortex 5 seconds to release cells
- Wait 15 minutes
- Remove brush with new tweezer

- Vortex 5 seconds

- Aliquots:

  - 450 µL to an empty 1.5 mL Eppendorf tubes (for MagNaPure DNA/RNA extraction and CLART HPV2 testing)
  - 1000 µL to empty CBD-tubes for BD Onclarity testing
  - (500 µL to HC2-analysis for the first 1008 samples)
  - Leftover material will serves as back-up material for re-testing and biobank material. stored in -20 0C
